# Dual-functional SERRS and fluorescent aptamer sensor for abscisic acid detection via charged gold nanorods

**DOI:** 10.3389/fchem.2022.965761

**Published:** 2022-08-15

**Authors:** Yanyan Zhang, Wei Li, Hao Zhang, Shun Wang, Xiaodong Li, Syed Muhammad Zaigham Abbas Naqvi, Jiandong Hu

**Affiliations:** ^1^ College of Mechanical and Electrical Engineering, Henan Agricultural University, Zhengzhou, China; ^2^ Henan International Joint Laboratory of Laser Technology in Agricultural Sciences, Zhengzhou, China; ^3^ College of Science, Henan Agricultural University, Zhengzhou, China; ^4^ State Key Laboratory of Wheat and Maize Crop Science, Zhengzhou, China

**Keywords:** abscisic acid, dual-functional sensor, fluorescence quenching and recovery, plant hormone, surface-enhanced resonance Raman spectroscopy

## Abstract

Abscisic acid (ABA) is a plant hormone, which plays an important role in plant growth, crop cultivation and modern agricultural engineering management. Accordingly, the detection of ABA content combined with new techniques and methods has become a more and more popular problem in the field of agricultural engineering. In this work, a SERRS and fluorescence dual-function sensor based on the fluorescence quenching and Raman enhancement properties of gold nanorods (AuNRs) was developed, and applied to the detection of plant hormone ABA. The dual-function reporter molecule Rhodamine isothiocyanate (RBITC) and complementary DNA (cDNA) were modified on AuNRs (AuNRs@RBITC@cDNA) as signal probes and aptamer modified magnetic nanoparticles (Fe_3_O_4_MNPs@Apt) as capture probes. Through the specific recognition of ABA aptamer and its complementary chains, an dual-function aptamer sensor based on SERRS and fluorescence was constructed. When ABA molecules were present in the detection system, the signal probes were detached from the capture probes due to the preferential binding between aptamer and ABA molecules. SERS signal of the reporter molecules appeared in the supernatant after magnetic separation, and it increased with the increase of ABA concentration. If the etching agent that can etch AuNRs was added to the supernatant, the AuNRs was etching disappeared, then the signal molecules fall off from the AuNRs, and the fluorescence signal intensity would recovered. The intensity of fluorescence signal also increased with the increase of ABA concentration. Thus, the quantitative relationship between ABA concentration and SERRS intensity and fluorescence intensity of signal molecules was established. The linear range of SERRS detection was 100 fM–0.1 nM, the detection limit was 38 fM; The linear range of fluorescence detection was 1 pM–100 nM, the detection limit is 0.33 p.m. The constructed dual-effect sensor was used in the recovery laboratory of real ABA samples, the recovery rate was up to 85–108%.

## 1 Introduction

Abscisic acid (ABA), also known as dormancy hormone, is widely distributed in a variety of higher plants (Wu et al., 2022). In addition to promoting plant leaf shedding, it can also promote plant bud to enter dormancy state (KozakiandAoyanagi, 2022), potato to form tuber (Tiwari et al., 2022) and other effects, and also inhibit the extension of plant cells (MusluandKadioglu, 2021). Due to the importance of ABA in botanical and agricultural engineering research, a variety of detection methods such as colorimetry (Zhou et al., 2012), local surface plasmon resonance (LSPR) (Wang et al., 2020), chromatography (Chen et al., 2010) and so on have been used to detect ABA and obtained good results. Among them, chromatography and ELISA (Jiraskova et al., 2009) are the most commonly used methods, but these two methods need professional technicians to operate, and are not suitable for most people in the field of agricultural engineering. Therefore, it is urgent to develop more novel, sensitive and convenient detection methods.

Surface enhanced Raman (SERS) is a phenomenon of significantly enhanced molecular scattering cross section adsorbed on noble metal nanostructures (Panikar et al., 2021). Further enhancement, known as surface enhanced resonance Raman scattering (SERRS), can be observed if the molecules attached to the metal surface contain chromophores of electron transitions consistent with the excitation wavelength, Therefore, SERRS is a progress based on SERS effect. With this feature, SERRS technology can even reach the level of single molecule detection (Kim et al., 2021), and the enhancement factor can reach 10^14^ to 10^15^ (Ansar et al., 2012). SERRS technology has been widely used in medicine (Moisoiu et al., 2021), biology (Bido et al., 2020), national defense (Huang et al., 2020), materials (AlessandriandLombardi, 2020) and other fields (Fan et al., 2020) (LinandHe, 2019), due to its advantages of high sensitivity, rapid detection, simple preprocessing and so on. The most important thing is that the application of SERRS can solve the defect that it is impossible for non-professionals to operate. Noble metal nanomaterials were one of the indispensable factors for the application of SERRS technology, noble metal nanomaterials with different shapes and structures showed different surface plasmon resonance characteristics and then showed different SERRS enhancement (Na et al., 2021). Due to its simple synthesis method and easy preservation, gold nanomaterials with different morphologies were the most common in SERRS technology application (Durucan et al., 2018). In recent years, gold nanorods (AuNRs) as the main representative of gold nanomaterials have attracted more and more attention in the field of biotechnology (Zhou et al., 2020). Compared with gold nanoparticles, anisotropic AuNRs exhibit higher stability, rapid electron transfer, and a wide spectral range associated with shape tunability of optical properties (HwangandTao, 2011). This makes AuNRs have the potential to be applied to SERS biological sensors (Yang et al., 2021) and other biological sensors such as the fluorescence biosensor (Liu et al., 2022).

With the increase of researchers’ attention to SERRS technology, more and more SERRS technology was combined with other detection technologies to construct dual-function detection technology. Compared with the single signal output method, the dual-function method can provide more response information, and the detection results of the two functions can be verified with each other (Hao et al., 2020), which greatly reducing the probability of false negative/positive detection, and the detection results were more convincing. For example, Luo *et al.* constructed a fluorescence and SERRS dual-mode biosensor to monitor telomerase activity in living cells. The SERRS and fluorescence signal intensities were controlled by changing the distance between the signal molecule Cy5 and AuNPs (Luo et al., 2022). Li *et al.* combined gold nanostars used for quenching fluorescence and enhancing Raman signal with DNA zyme to form nanoprobes to developed a new type of dual-mode nanoprobes, which including “on” fluorescence and “off” SERRS, could be used for the determination of sensitive and selective Ca^2+^. The nanoprobes combined the advantages of SERRS and fluorescence, and had good versatility in a variety of cell lines, contributing to a better understanding of the role of Ca^2+^ in cellular pathways (Li et al., 2021). These studies showed that the dual-mode fluorescence SERRS detection method based on dual-function was able to cope with different detection conditions and had specific recognition ability, which attracted more and more attention *in vitro* and *in vivo* biological detection. However, the study on the application of fluorescence SERRS dual-mode detection system for plant hormones has not been seen yet.

**SCHEME 1:**
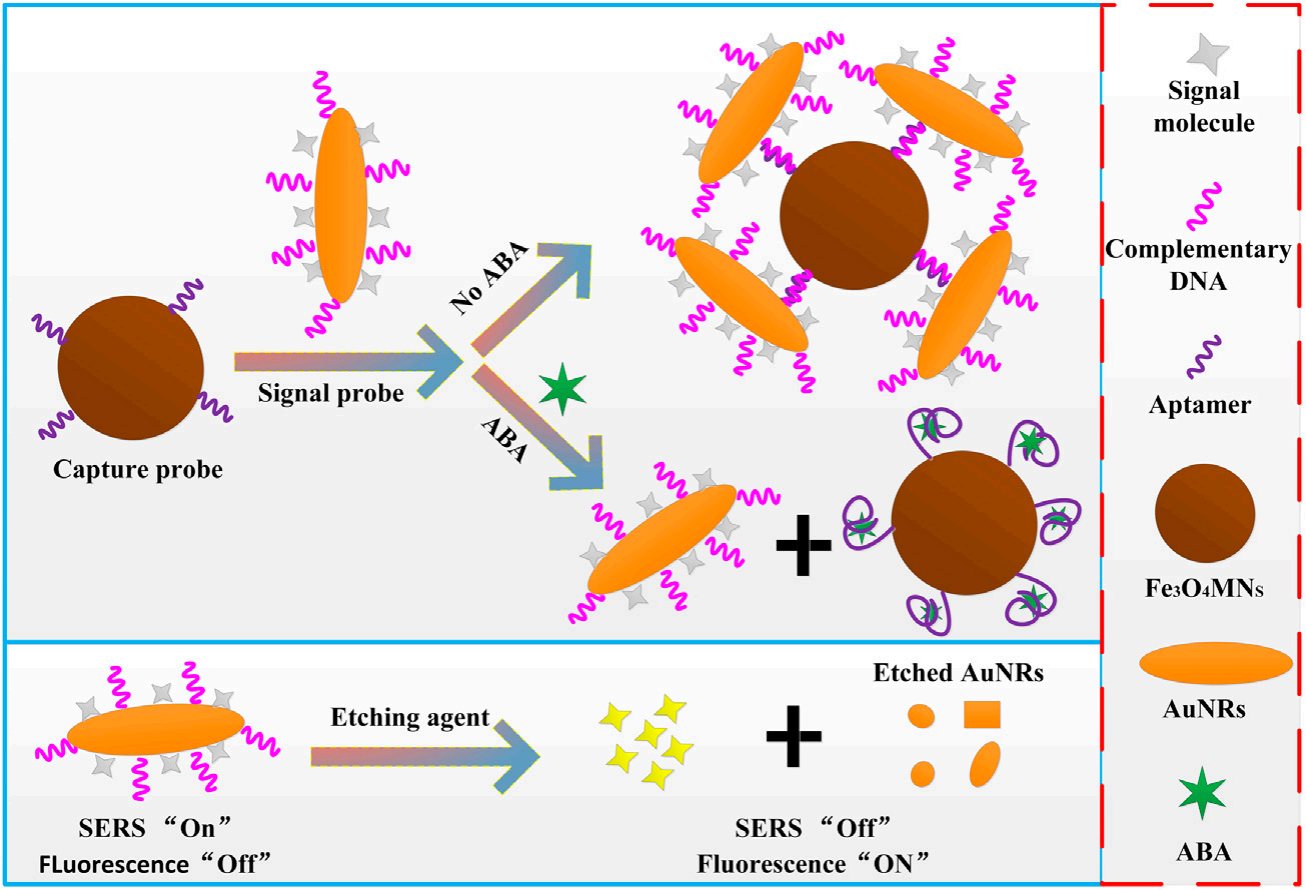
Schematic illustration of ABA detection by using the dual-functional sensor.

In this work, a new fluorescence and SERRS dual-function detection method for ABA was proposed (Scheme 1). First, the dual-functional signal molecules with Raman and fluorescence properties RBITC were modified on AuNRs. Because of the resonance between the Raman laser (532 nm) and the strongest absorption peak (547 nm) of the RBITC molecule. Once RBITC was adsorbed on AuNRs, RBITC showed strong (SERRS) signal by virtues of the plasma resonance characteristics of AuNRs and coupling between SERRS photons and plasmons. In addition, due to the light quenching of AuNRs for fluorescent molecules, RBITC binding with AuNRs can show measurable fluorescence reduction. Therefore, the SERRS intensity and fluorescence intensity before and after the RBITC molecules were combined with AuNRs were used to detect the molecular concentration of plant hormone ABA indirectly, and the linear relationship between the ABA concentration and the SERRS intensity and fluorescence intensity of probe molecule RBITC were established. The dual-functional sensor developed by this method showed good sensitivity and anti-interference in ABA detection. In addition, the main function of Fe_3_O_4_MNPs was separation and enrichment, which greatly simplifies the detection process. Therefore, the proposed dual-functional aptamer sensor can be used as a potential tool for the detection of plant hormones to fill the gap of the simultaneous detection of SERRS and fluorescence.

## 2 Experimental section

### 2.1 Materials and instruments

Chlorauric acid (HAuCl_4_·4H_2_O), trisodium citrate (C_6_H_5_Na_3_O_7_), cetyl trimethyl ammonium bromide (CTAB), sodium borohydride (NaBH_4_), silver nitrate (AgNO_3_), ascorbic acid (AA), Hydrochloric acid (HCl), rhodamine B isothiocyanate (RBITC) were purchased from Sigma-Aldrich (United States, analytical pure). Potassium ferricyanide (Potassium ferricyanide, K_3_FeC_6_N_6_, AR,≧99.5%), Potassium iodide (KI, AR,≧99%) were purchased from Shanghai Maclin Biochemistry Co., LTD. (Shanghai, China). Aptamer (Apt) and aptamer complementary chain sequences (cDNA) were shown in Supplementary Table S1, synthesized by Sangong Bioengineering (Shanghai) Co., LTD. The existing oligonucleotide chains were heated at 95°C for 5 min and then cooled rapidly in an ice bath.

The fluorescence signals were collected by a fluorescence spectrophotometer (Fluorolog3, HORIBA, United States), in the process of spectral acquisition, a 391 nm laser was used to the excitation light. The SERRS signals were collected by a Raman microscopic system (Pioneer Technology Co. LTD., Beijing, China). In the process of spectral acquisition, a 532 nm laser was used to the excited source, a 100 × objective lens was used to focus the laser, the scanning range is 800–1800 cm^−1^, and the integral time is 10 s. The UV-vis spectra were recorded by the UV-vis spectrophotometer (Nanjing Feiler Instrument Co. LTD., Nanjing, China). The transmission electron microscope (TEM) images were taken on a transmission electron microscope (JEM-1400 Plus, JEOL Ltd., Tokyo, Japan) operating at 120 kV. Zeta potentials was obtained by particle and molecular size analyzer (Zetasizer Nano ZS, Malvern Instruments Ltd. Melvin, United Kingdom).

### 2.2 Construction of signal probes

Signal probes construction was divided into three steps: 1) Synthesis of AuNRs; 2) The formed of AuNRs@cDNA by modifying the complementary chains (cDNA) of ABA aptamer containing sulfhydryl group on AuNRs; 3) The dual-functional signal probes AuNRs@cDNA@RBITC were formed by modifying Raman and fluorescence double functional molecules RBITC on AuNRs@cDNA.

The synthesis of AuNRs has been slightly modified by referring to the “tips” literature (Scarabelli et al., 2015). Firstly, synthesized the “gold seed” solution. Water bath at 30°C condition, 4.7ml, 100 mM CTAB solution was putted into 20 ml glass bottle, 50 µl 1% chlorauric acid solution was added, slow magnetic stirring for 3 min, until the solution clear, 300 μl, 10 mM ice NaBH4 solution added quickly and stirred vigorously. After 15 s, the solution turned bright brown, the gold seed solution was successfully synthesized, and was placed in a water bath and let stand. Secondly, synthesized “growth” solution. 200 µl, 1% HAuCl_4_·4H_2_O solution was added to 10 ml, 100 mM CTAB solution and agitated slowly for 5 min, 120 μl, 10 mM AgNO_3_ solution was added, agitated slowly for a few seconds, then 100 μl, 100 mM AA solution was quickly added, After stirring vigorously for 5 s, the solution becomes colorless. Finally, 24 µl gold seed solution was added to the growth solution and stirred vigorously for 2 min. After standing in water bath at 30°C for 12 h, the gold seeds grew into gold nanorods.

AuNRs@cDNA was formed by modifying the cDNA on AuNRs. 10 µl 100 µM cDNA solution was added in 1 ml AuNRs, incubated at room temperature for 2 h, centrifuged at 8,000 rpm for 10 min, and the precipitate was dispersed in 1 ml water to form AuNRs@cDNA.

The signal probes AuNRs@cDNA@RBITC were formed by modifying dual-functional molecules RBITC on AuNRs@cDNA. 10 µl, 500 µM RBICT solution was added in 1 ml AuNRs@cDNA solution, after incubation for 2 h, the supernatant was removed by centrifuged at 8,000 rpm for 10 min, and the precipitate was dispersed in 1 ml water to form AuNRs@cDNA@RBITC signal probes.

### 2.3 Construction of capture probes

The capture probes were constructed by modifying aptamer of ABA (aptamer modified biotin) on Fe_3_O_4_MNPs (Fe_3_O_4_MNPs modified streptavidin). Fe_3_O_4_MNPs were purchased at a concentration of 50 mg/ml and diluted to 2.5 mg/ml in ultrapure water as shock solution. 10 µl, 100 µM aptamer solution was added to 1 ml Fe_3_O_4_MNPs, vortex shock for 1 min, magnetic separation was performed after standing at room temperature for 2 h, the precipitate was dispersed in 1 ml water to form Fe_3_O_4_MNPs@Apt capture probes. The UV-Vis spectra was recorded before and after the Fe_3_O_4_MNPs modified aptamer to characterize the successful construction of the capture probes.

### 2.4 Construction of dual-functional aptamer sensor for abscisic acid quantitative detection

In the process of sensor construction, some factors inevitably affect the performance of sensor, such as the ratio between the two probes, the competition time between ABA and aptamer, etc. Therefore, the optimized volume ratio of signal probes to capture probes before ABA detection was 1.75:1, the competitive binding time between ABA and aptamer was 75 min, and the etching time of AuNRs was 5 min.

Under the optimized conditions, the constructed sensor was used for quantitative detection of ABA. 50 µl different concentrations (1 × 10^−13^ M, 1 × 10^−12^ M, 1 × 10^−11^ M, 1 × 10^−10^ M, 1 × 10^−9^ M, 1 × 10^−8^ M, 1 × 10^−7^ M, 1 × 10^−6^ M) of ABA solution were added to 300 µL sensor solution, after 75 min of room temperature competitive reaction, magnetic separation was carried out and the supernatant was used to SERRS detection. Then, 100 µl etching agent was added to supernatant and its fluorescence emission spectra were recorded after 5 min. The limit of detection (LOD) of ABA was calculated using the classic formula 3S_b_/b, where S_b_ is the standard deviation of the Raman intensity of the blank sample at 1,654 cm^−1^ Raman shift or fluorescence intensity of the blank sample at emission wavelength 639 nm b is the slope of the SERS or fluorescence drawn standard curve.

### 2.5 Selectivity evaluation

The growth and development process of plants is the result of the joint action of various plant hormones and nutrients, so the detection of ABA may be affected by other plant hormones such as auxin, gibberellin, as well as nutrients such as β-carotene (β-car) and vitamin C (VC) in the samples of plant hormones. In order to test the specificity of the sensor for ABA detection, 1 mM of three kinds of plant hormone auxin, gibberellin, salicylic acid as the target molecules were added into the sensor solution, after incubation for 75 min at room temperature. The supernatant was used for SERRS detection after magnetic separation, and fluorescence detection was carried out after etching agent was added into the supernatant for 5 min. The results were compared with those of ABA.

### 2.6 Abscisic acid detected in real sample

ABA samples were extracted from mature wheat leaves, and the extraction method of real samples was referred to (Zhang et al., 2021). The concentration of extracted real ABA samples was calibrated using the HPLC method. Then the blank recovery of real ABA samples was tested using the constructed dual-function sensor.

## 3 Results and discussions

### 3.1 Characterization of the signal probes

**FIGURE 1:**
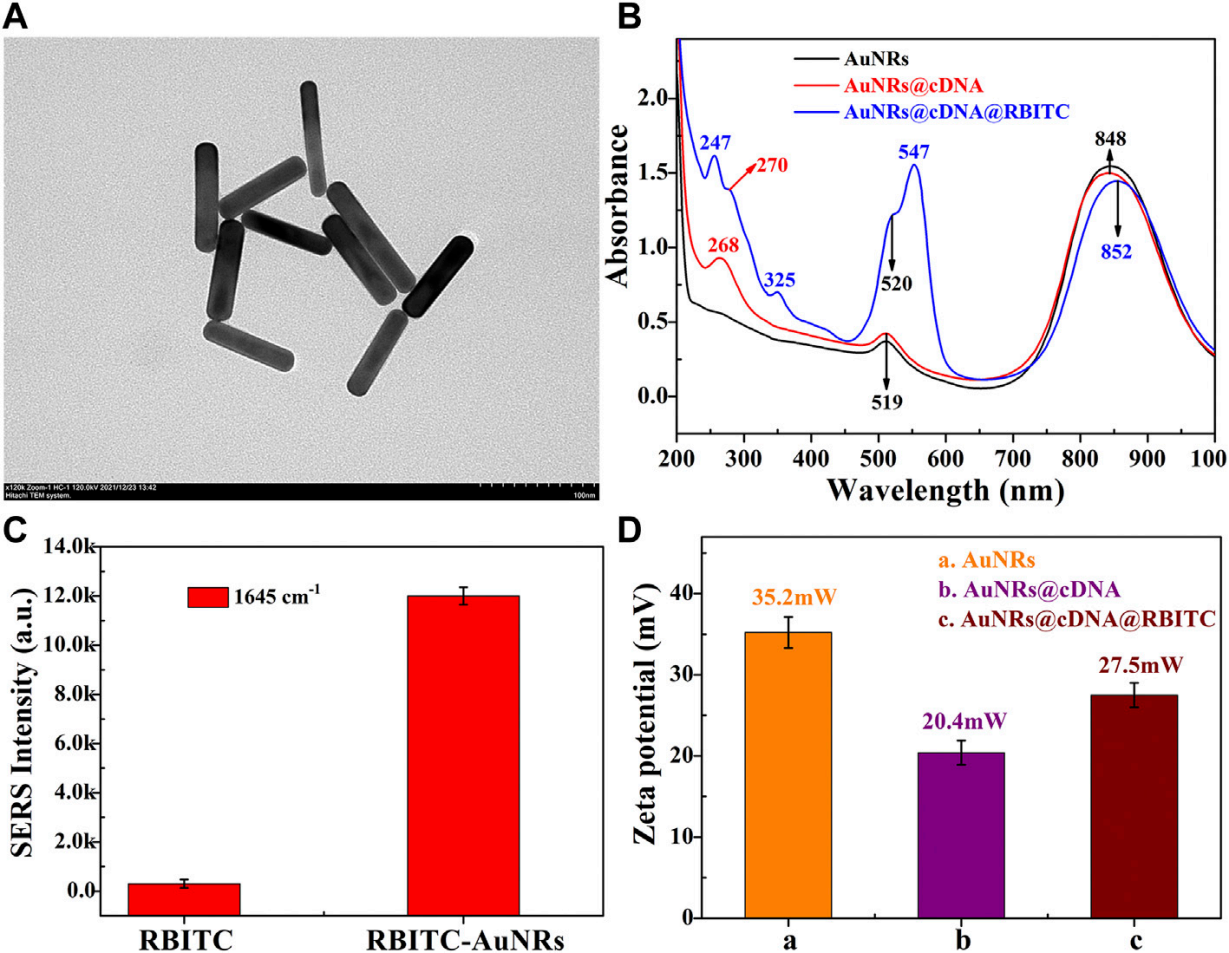
Characterization of signal probes. **(A)** TEM of AuNRs; **(C)** The Raman intensity of the characteristic peak at 1645cm^−1^ before and after RBITC binding AuNRs; The changes in **(B)** UV-Vis spectra; **(D)** ZETA potential.

The cDNA of ABA aptamer and the dual-functional signal molecules RBITC were modified on AuNRs to obtain signal probes. The shape of AuNRs was regular and uniform (Figure 1A), with an average size of 75 × 15 nm (Supplementary Figure S1). In combination with UV-Vis spectra of AuNRs (Figure 1B B, black line), it can be seen that the transverse peak of the synthesized AuNRs was 519 nm and the longitudinal peak was 848 nm. TEM and UV-Vis spectra can indicated the successful synthesis of AuNRs. Figure 1C shows the intensity of SERRS spectrum before and after the combination of RBITC molecule and AuNRs, which clearly shows that the SERRS intensity before and after the combination of RBITC and AuNRs was than ten times different, which indicated the synthesized AuNRs have good fluorescence quenching and SERRS enhancement. The red line in Figure 1B was the UV-Vis spectrum of cDNA modified on AuNRs. In addition to the absorption peak of AuNRs, a clear absorption peak of cDNA was located at 268 nm. The blue line in Figure 1B shows the UV-Vis absorption spectrum of AuNRs after modification of cDNA and signal molecule RBITC. Except for the SPR peaks of AuNRs and cDNA, the absorption peak of RBITC was 247 nm, 325 nm, and 547 nm clearly visible. Interestingly, the maximum SPR peak of RBITC at 547 nm almost coincides with the excitation wavelength of Raman instrument at 532nm, so SERRS can occur during the detection process. This could also explain to why RBITC molecules combine with AuNRs to produce super strong Raman signals (Figure 1C). Combined with the changes of zeta potential data during the formation of the signal probes, as shown in Figure 1D, the AuNRs prepared by seed growth method were positively charged with a potential of 35.2 mV. The HS -DNA was negatively charged, and the potential of the AuNRs decreased to 20.4 mV after the DNA chain was modified. RBITC aqueous solution is positively charged, and the sulfhydryl group in its molecular structure forms covalent bonds with AuNRs, and the potential was 27.5 mV after modification on AuNRs. The change of potential was consistent with UV-Vis spectrum, indicating that the signal probes were successfully constructed.

**FIGURE 2:**
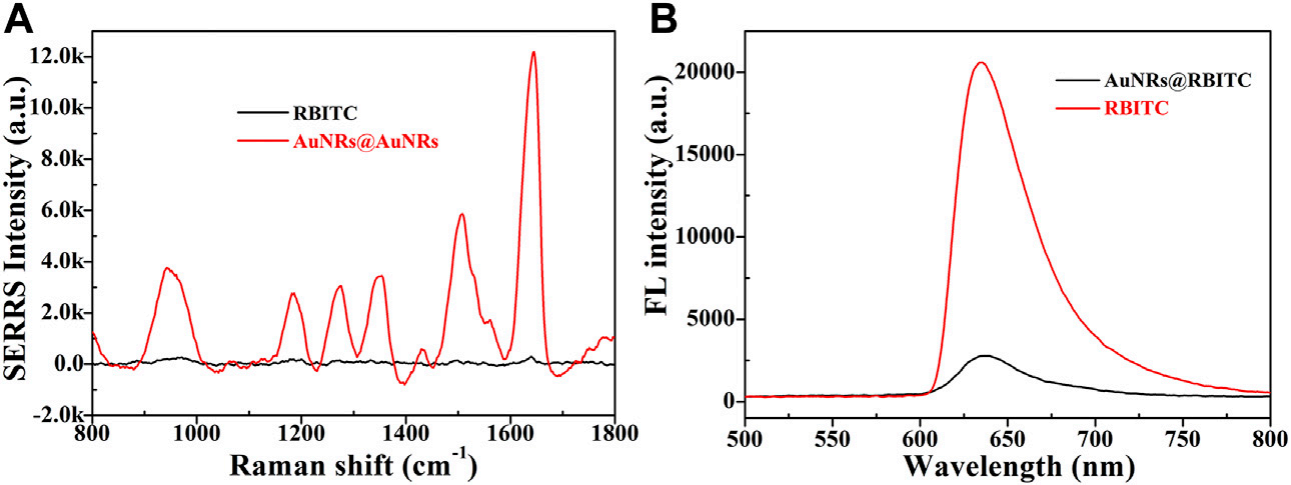
The changes in SERRS intensity **(A)** and fluorescence intensity **(B)** of RBITC signal molecule before and after binding AuNRs.

In order to further verify the feasibility of the experimental scheme, SERRS and fluorescence spectrum of AuNRs before and after binding with signal molecule RBITC were tested. As shown in Figure 2, before combining with AuNRs, the signal molecule RBITC has strong fluorescence, while its Raman signal was weak. After combining with AuNRs, because of AuNRs had fluorescence quenching and SERS enhancement, which greatly enhanced the SERRS intensity and weakened the fluorescence intensity of RBITC. This result combined with UV-Vis spectrum and ZETA characterization result, it can be indicated the signal probes was successful constructed.

### 3.2 Characterization of the capture probes

**FIGURE 3:**
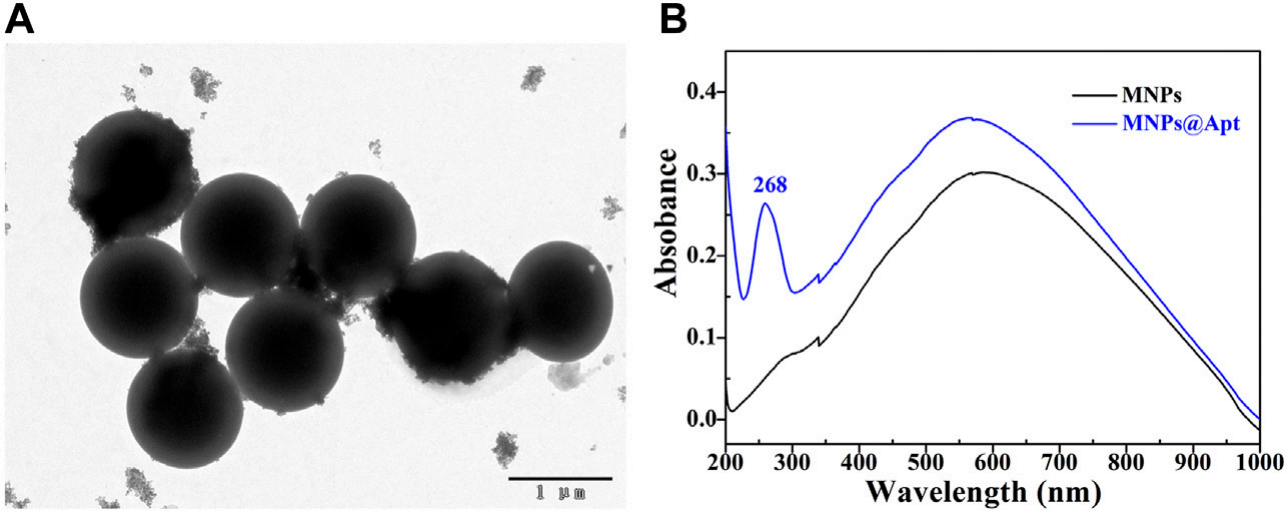
Characterization of capture probes. **(A)** TEM of Fe_3_O_4_MNPs; **(B)** The UV-vis spectra before and after aptamer binding Fe_3_O_4_MNPs.

Streptavidin modified 500 nm Fe_3_O_4_MNPs were purchased from Sangon Biotechnology Co., LTD (Shanghai). Its TEM was shown in Figure 3A, indicating that Fe_3_O_4_MNPs have regular morphology. The aptamer successful assembled on the Fe_3_O_4_MNPs was proven by UV-Vis spectra, compared with the UV-vis spectrum of Fe_3_O_4_MNPs (Figure 3B black line), the UV-vis spectrum of Fe_3_O_4_MNPs@Apt (Figure 3B blue line) have obvious DNA absorption peak at 268 nm, indicating that the aptamer has been successfully modified on Fe_3_O_4_MNPs to form capture probes. Furthermore, Our previous work also demonstrated that modifying aptamers on magnetic nanoparticles does not significantly affect its magnetism (Li et al., 2010). Therefore, the modified aptamer magnetic nanoparticles still have good paramagnetism.

### 3.3 Optimization of the dual-functional aptamer sensor

**FIGURE 4:**
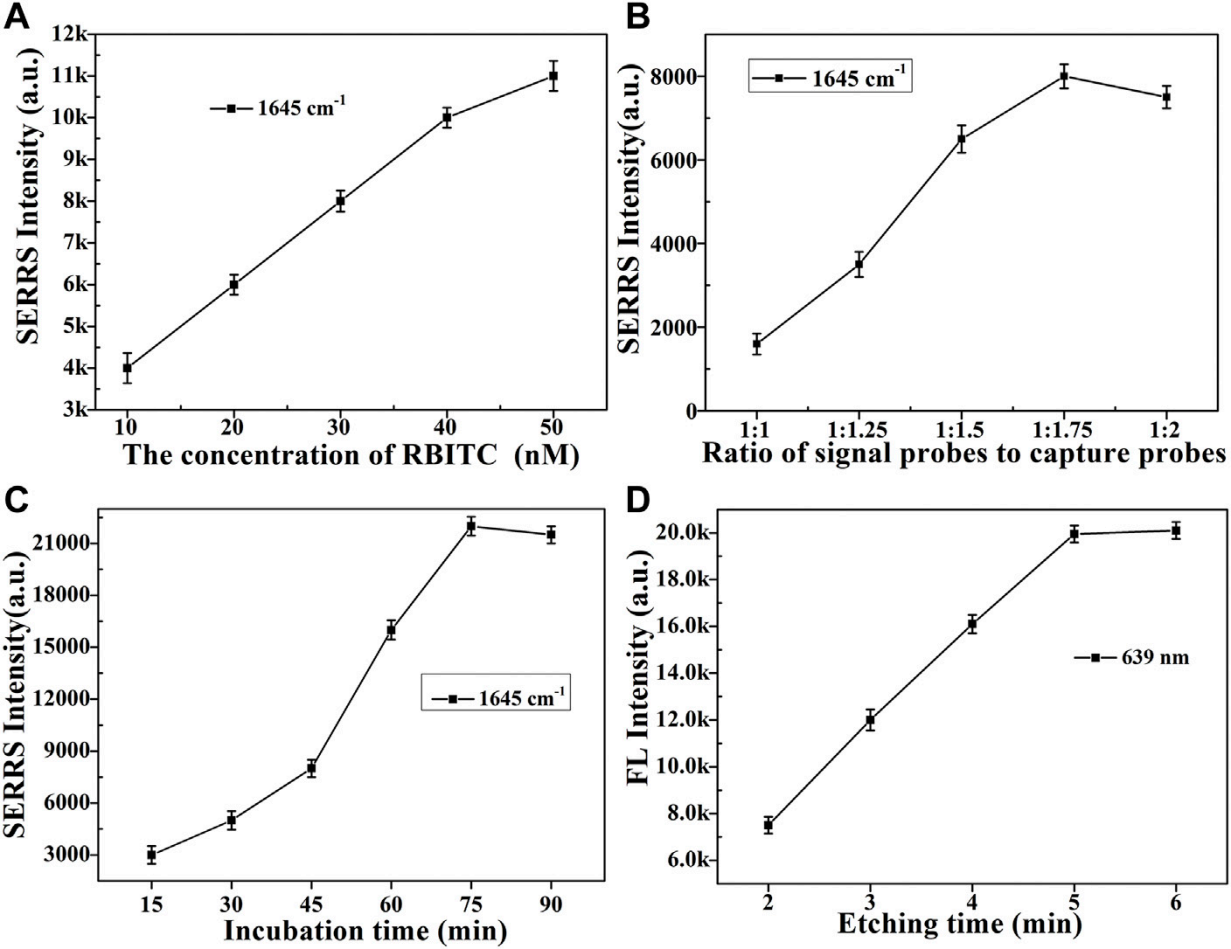
Optimization of various factors. SERS intensity of RBITC at 1,645 cm^−1^ of **(A)** different concentrations of RBITC were modified on the AuNRs@cDNA; **(B)** different volume ratios (1:1, 1:1.25, 1:1.5, 1:1.75, 1:2) of signal probes and capture probes; **(C)** different incubation times (15, 30, 45, 60, 75, 90 min) of ABA (1 × 10^−8^ M) added into sensor solution; **(D)** FL intensity of RBITC at 639 nm of the sensor solution after etched.

During the successful construction of dual-functional aptamer sensor, the performance of the sensor inevitably affected by some factors. Therefore, these potential factors were investigated. Firstly, the concentration of signal molecule RBITC was optimized (Figure 4A and Supplementary Figure S2). As the concentration of signal molecule RBITC increased, the SERRS intensity of the obtained signal probes gradually increased. When the final concentration of RBITC was 50 nM, the degree of increase in SERRS intensity decreased compared with the previous concentrations, indicating the surface of AuNRs@cDNA was about to be saturated. Therefore, in order to ensure the sensitivity of the constructed SERS dual-functional aptamer sensor, the concentrations of the added signal molecules were 10 μl, 5 µM for each 990 µl AuNRs@cDNA solution.

**FIGURE 5:**
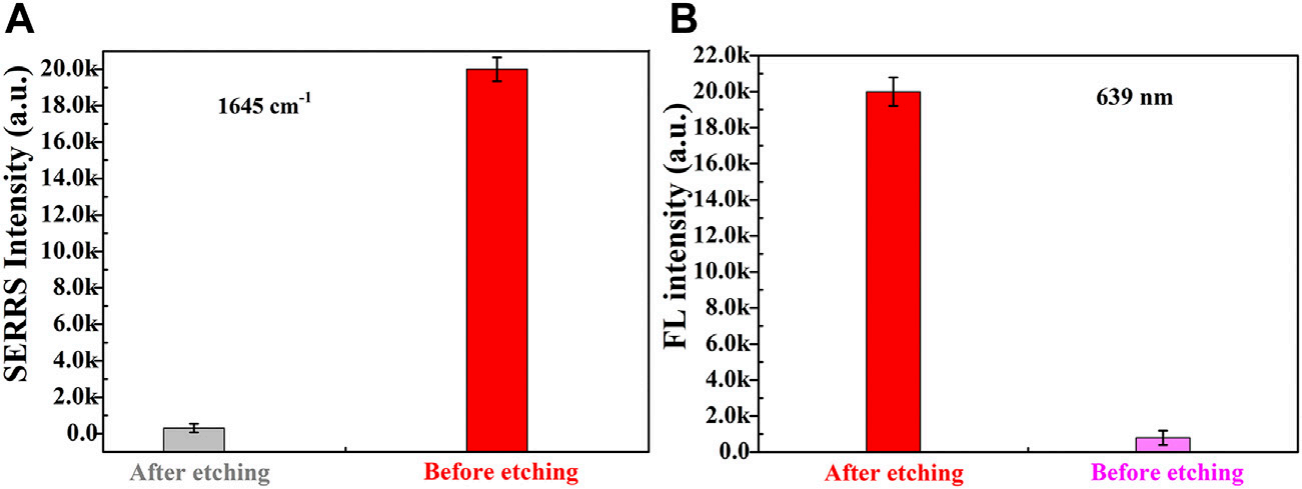
The **(A)** SERRS and **(B)** fluorescence intensities of dual-functional aptamer sensor solution before and after etched..

Secondly, the size of Fe_3_O_4_MNPs was relatively larger than that of AuNRs, in order to construct a dual-functional aptamer sensor with high signal stability and sensitivity, the volume ratio of the two probes was optimized (Figure 4B and Supplementary Figure S3). The signal probes and capture probes with different volume ratios (1:1, 1.25:1, 1.5:1, 1.75:1, 2:1) were mixed, after incubation at room temperature for 1 h, the supernatant was removed by magnetic separation, and the precipitate was dispersed in the same volume of water. 10 µl of sensor solution was dropped on the silicon wafer, and SERRS detection was conducted after drying at room temperature. With the increase of the ratio of signal probes to capture probes, more signal probes were captured on capture probes, and more signal probes were obtained after magnetic separation, resulting in higher SERS intensity. When the ratio of signal probe to capture probe exceeded 1.75:1, SERRS intensity did not increase significantly, indicating that the signal probes at this time occupied all aptamers on the capture probes and the generated signal reached the maximum, so the ratio of signal probe to capture probe was 1.75:1. If sufficient etching agent was added to the constructed aptamer sensor solution at the same time, the drastic reduction of SERS intensity of signal molecules and the recovery of fluorescence intensity could be clearly observed. As shown in Figure 5, which further indicated the successful construction of the dual-functional aptamer sensor.

Thirdly, when a certain concentration of ABA solution was added for detection after the construction of the dual-function aptamer sensor, ABA molecules would preferably bind to the aptamer, leading to the separation of the signal probes from capture probes. Therefore, it is necessary to optimize the competition time after the addition of ABA solution. As can be seen from Figure 4C and Supplementary Figure S4, With the increase of competitive incubation time, the SERS intensity of signal molecules RBITC in the supernatant increased, indicating that the more signal molecules separated from the capture probes. When the incubation time reached 75 min, the SERRS intensity in the supernatant reached the maximum. If the incubation time continued to increase to 90 min, the SERRS intensity of signal molecules almost unchanged. Therefore, 75 min was chosen as the competitive reaction time in the later ABA detection.

Lastly, when enough concentration of etching agent was added sensor solution, all AuNRs can be etched disappear, signal molecules dissociated from the AuNRs, the fluorescence intensity of signal molecules will be recovered. 100 µl etching agent was added into 1 ml dual-function aptamer sensor solution, and fluorescence intensity was detected after shocked, as shown in Figure 4D and Supplementary Figure S5. With the increase of etching time, more AuNRs was etched away, and the fluorescence intensity of signal molecules recovered. When the etching time was more than 5 min, the fluorescence intensity did not increase, indicating that the etching of AuNRs was completed. Therefore, 5 min was chosen as the etching time in the later ABA detection. The LSPR changes of the signal probes during etching in Supplementary Figure S6 and TEM image of the signal probes after etched in Supplementary Figure S7 in the revised supplementary material can indicate that the AuNRs were almost etched away.

### 3.4 The detection of abscisic acid based dual-functional aptamer sensor

**FIGURE 6:**
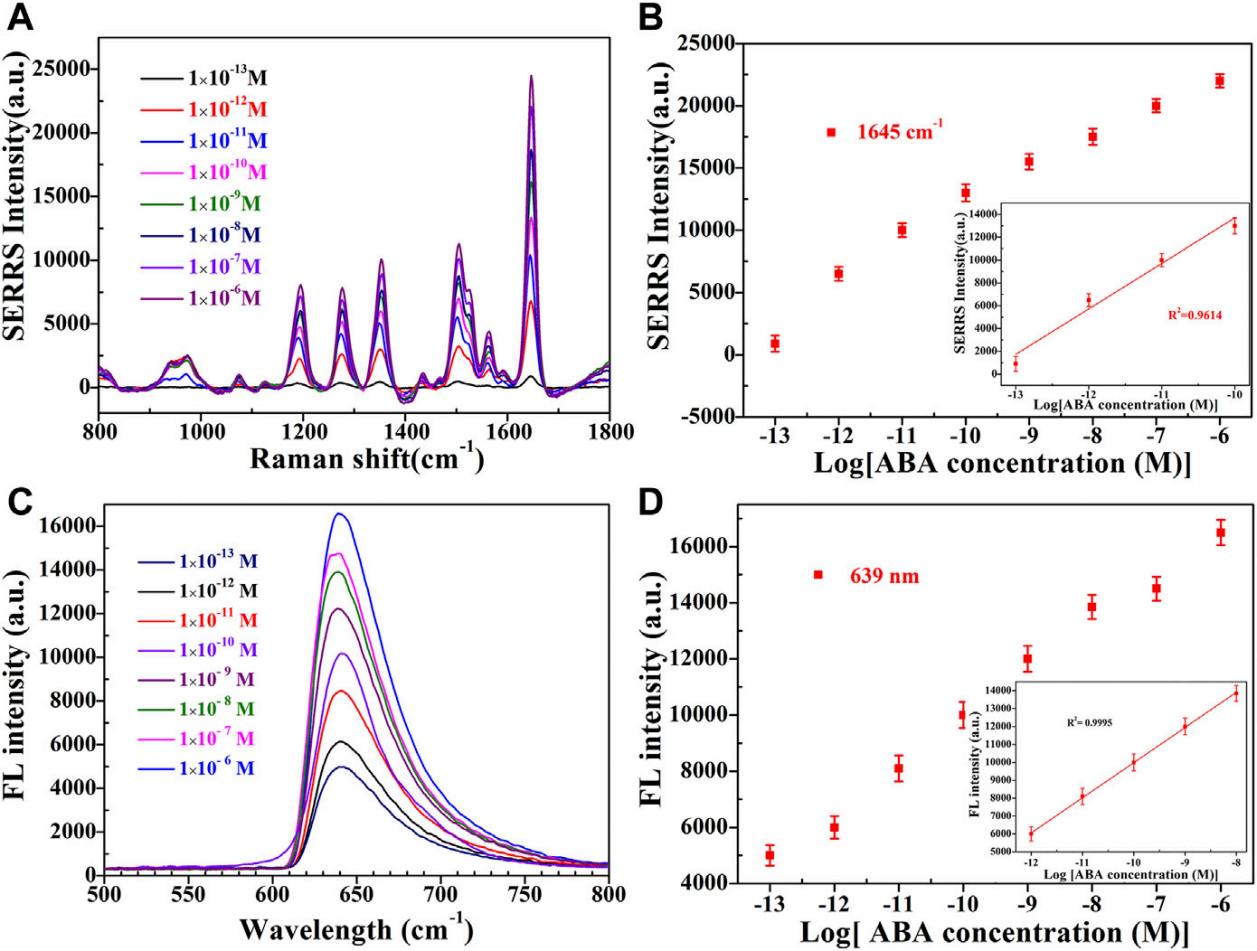
**(A)** SERRS and **(C)** Fluorescence emission spectra of sensor in the presence of different concentrations (1 × 10^−13^ M, 1 × 10^−12^ M, 1 × 10^−11^ M, 1 × 10^−10^ M, 1 × 10^−9^ M, 1 × 10^−8^ M, 1 × 10^−7^ M, 1 × 10^−6^ M, from bottom to top) of ABA. The linear plots of the **(B)** SERRS intensity and **(D)** The fluorescence intensity versus the ABA concentration in the logarithmic range. Error bars show the standard deviation of three repeated experiments.

Under the optimized conditions, the constructed dual-functional aptamer sensor was used for quantitative detection of ABA. Different concentrations of ABA solution were added to 300 µl sensor solution, and after 75 min of room temperature competitive reaction, the supernatant was taken for SERS detection after magnetic separation, 100 µl etching agent was added to the supernatant and fluorescence detection was performed 5 min later. Figures 6A,B showed that SERRS intensity of signal molecule RBITC increased with the increase of ABA concentration. SERRS intensity showed a linear relationship within the logarithmic range of 100 fM-0.1 nM. The linear regression equation of ABA detection was y = 402x+35,566, the corresponding R^2^ was 0.9614, and the calculated LOD was 38 fM. Figures 6C,D showed that fluorescence intensity of signal molecule RBITC increased with the increase of ABA concentration. Fluorescence intensity showed a linear relationship within the logarithmic range of 1 pM–100 nM. The linear regression equation of ABA fluorescence detection was y = 1962x+29,607, the corresponding R^2^ was 0.9995, and the calculated LOD was 0.33 p.m. The LOD of SERS detection was lower than ELISA (0.01p.m.) (Jiraskova et al., 2009), and the LOD of fluorescence detection was lower than HPLC (4.43 nM) (Chen et al., 2010). Due to the good fluorescence quenching ability of gold nanorods, the sensitivity of this method was comparable to some reported methods, indicating that this method has a high potential for ABA detection (Table 1).

**TABLE 1:** Comparison of the detection results of the developed method with some existing methods for abscisic acid detection.

Number	Method	Detection range	LOD	References
1	HPLC	4.43–14.2 nM	4.43 nM	Chen et al. (2010)
2	Electrochemical immunoassay	10–5,000 ng/ml	1 ng/ml	Li et al. (2010)
3	ELISA	0.01–10 p.m.	0.01 p.m.	Jiraskova et al. (2009)
4	Fluorescence	100–600 nM	100 nM	Sharmah et al. (2019)
5	LSPR	1 nM-10 μM	0.51 nM	Wang et al. (2020)
6	SERS	1 fM-10 nM	0.67 fM	Zhang et al. (2022)
7	SERS + Fluorescence	**SERS:** 0.1 pM-0.1 nM	0.38 fM	**This work**
		**FL:** 1 pM-10 nM	0.33 p.m.	

### 3.5 The selectivity of the sensor

**FIGURE 7:**
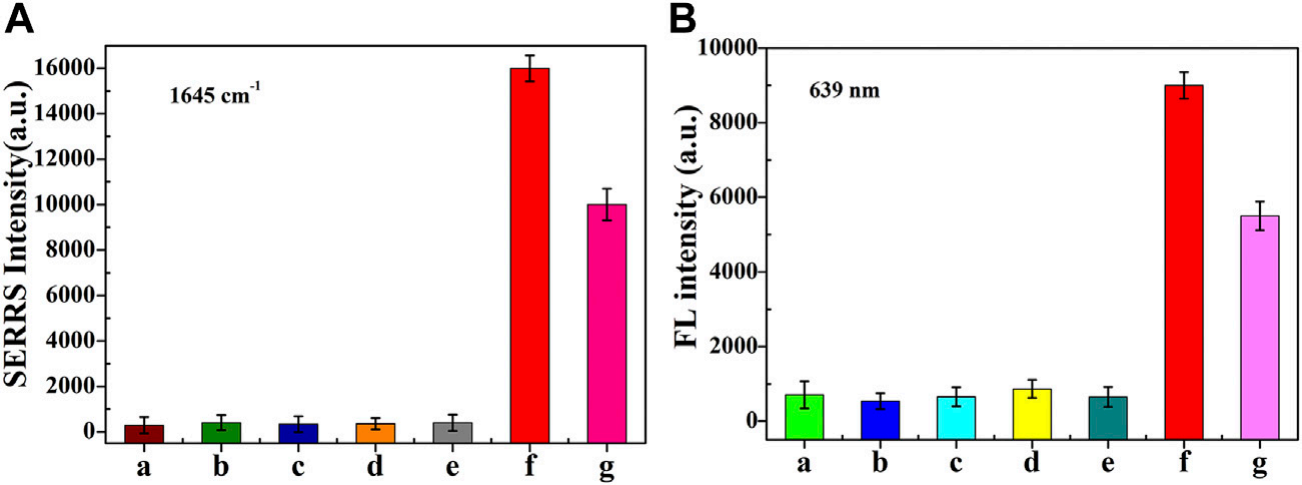
The SERRS **(A)** and fluorescence intensity **(B)** of dual-functional aptamer sensor for a. blank , b. GA3 , c. Auxin, d. β-Car, e. VC, f. ABA and g. The mixture of them. The concentration of ABA was 2 nM, while other interfering molecules were 2 µM. Error bars show the standard deviation of three repeated experiments.

As shown in Figures 7A,B, under the condition of ABA concentration of 2 nM, the fluorescence intensity and SERRS intensity were obviously stronger, while under the condition of other interfering substances concentration of 2 μM, the fluorescence and SERS intensity were almost the same as the blank condition. It is worth noting that the coexistence of interfering substances led to a slight decrease in the intensity of ABA fluorescence and SERRS signal, which may be due to the decrease in the concentration of ABA caused by the mixing of interfering substances. The interference experiment showed that the method presented in this paper had a good selectivity for ABA detection.

### 3.6 Abscisic acid determination in real wheat leaves

The feasibility of the proposed dual-functional sensor for ABA detection in real wheat samples was further discussed by addition and recovery experiments. As shown in Table 2, the final concentrations of the real samples were 2 μM, 0.5 and 0.2 µM respectively, the detected concentrations of SERS were 2.1, 0.54 and 0.19 µM respectively, good recoveries were 105%, 108%, 95%, and the maximum RSD was 8.75%. In addition, the detected concentrations of fluorescence were 2.15, 0.53 and 0.17 µM respectively, good recoveries were 107.5%, 106 and 85%, the maximum RSD was 9.57%. Addition and recovery experiments indicating that the proposed dual-functional sensor platform can be used for ABA detection in the real samples.

**TABLE 2:** The recovery experiments for abscisic acid detection in real wheat samples (n = 5).

Samples	Added (µM)	Found (SERS)	R (µM)ecovery (%)	RSD (%)	Found (FL)	R (µM)ecovery (%)	RSD (%)
1	2	2.1	105	6.71	2.15	107.5	7.65
2	0.5	0.54	108	4.95	0.53	106	5.36
3	0.2	0.19	95	8.75	0.17	85	9.57

## 4 Conclusion

In conclusion, a novel and reliable fluorescence and SERRS dual-functional aptamer sensor was developed based AuNRs modified with signal molecule RBITC as dual-functional signal probes for detecting plant hormone ABA. Under the optimal conditions, the SERRS and fluorescence intensity of signal probes increased proportionally with the increase of ABA concentration in the range from 1 × 10^−13^ M to 1 × 10^−6^ M, with a detection limit of 38 fM and 0.33 p.m. respectively. In addition, the proposed sensor exhibited a good selectivity for ABA detection. Moreover, satisfactory results were obtained when the assay was applied for ABA detection in wheat real samples, suggesting that the proposed sensor has potential application value for ABA detection in complex plant samples. However, studies on the detection of ABA by dual-function aptamer sensor is still in the laboratory research stage. For the detection of real samples, ABA still needs to be extracted from wheat plants, which is a big obstacle to the realization of online detection of ABA. Therefore, the research group will work towards the combination of SERS biosensor technology with a wearable device which can quickly extract ABA from plants.

## Data Availability

The original contributions presented in the study are included in the article/Supplementary Material, further inquiries can be directed to the corresponding author.
