# Supplementary material for: Dual-functional SERRS and fluorescent aptamer sensor for abscisic acid detection via charged gold nanorods
Source: Front Chem. 2022 Aug 15;10:965761. doi: 10.3389/fchem.2022.965761 (PMC9420979; doi:10.3389/fchem.2022.965761)
Supplement: Supplementary file 1 [file DataSheet1.docx]

Supplementary Material

**Supplementary Table 1.** Aptamer sequences used in the experiment

| **Name** | **Sequences (5′-3′)** |
| --- | --- |
| Apt | Bio- ATG GGT TAG GTG GAG GTG GTT ATT CCG GGA ATT CGC CCT AAA TAC GAG CAA C |
| cDNA | SH- ATA ACC ACC TCC ACC TAA CCC AT |


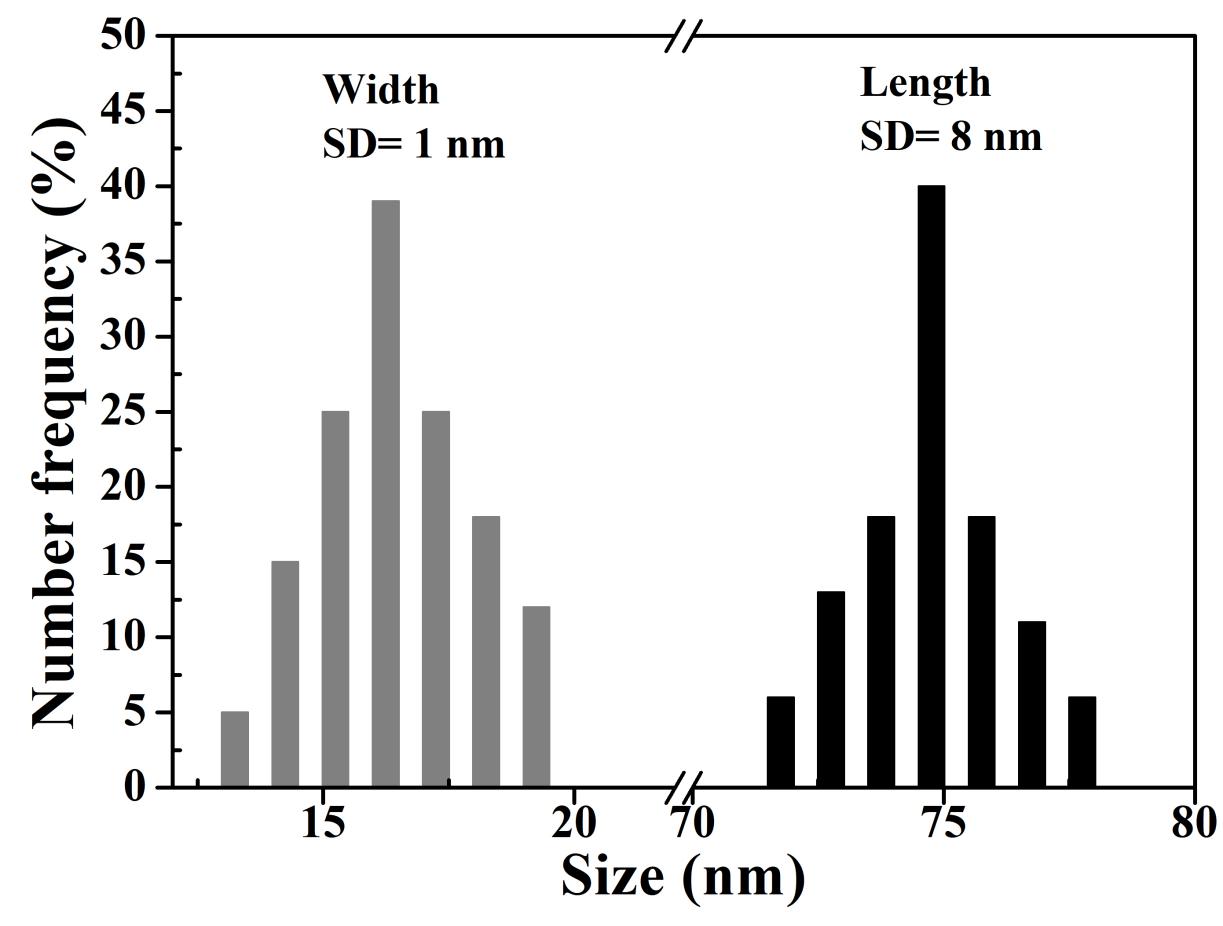


**Supplementary Figure 1.** Article size distribution of AuNRs.

##
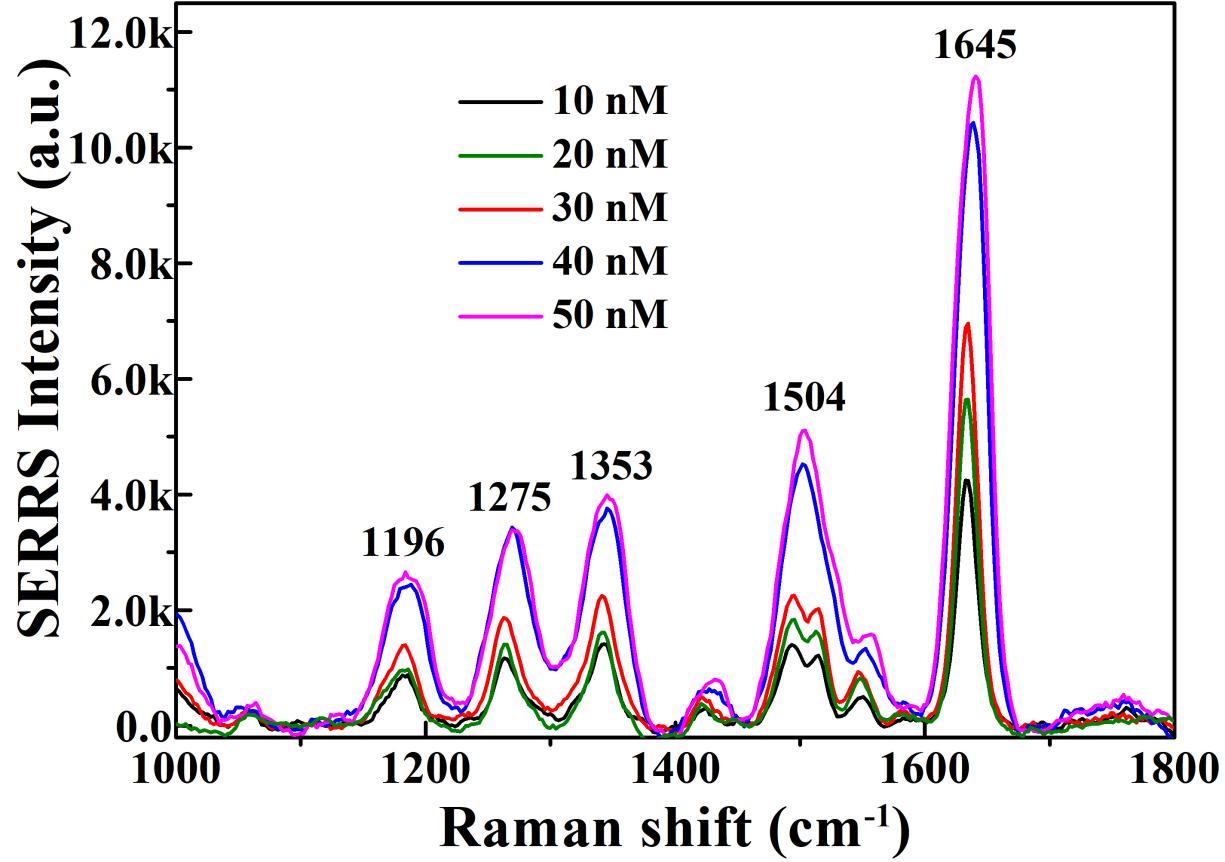


**Supplementary Figure 2.** SERS intensity of RBITC at 1645 cm^-1^ of different concentrations of RBITC were modified on the AuNRs@cDNA.

**
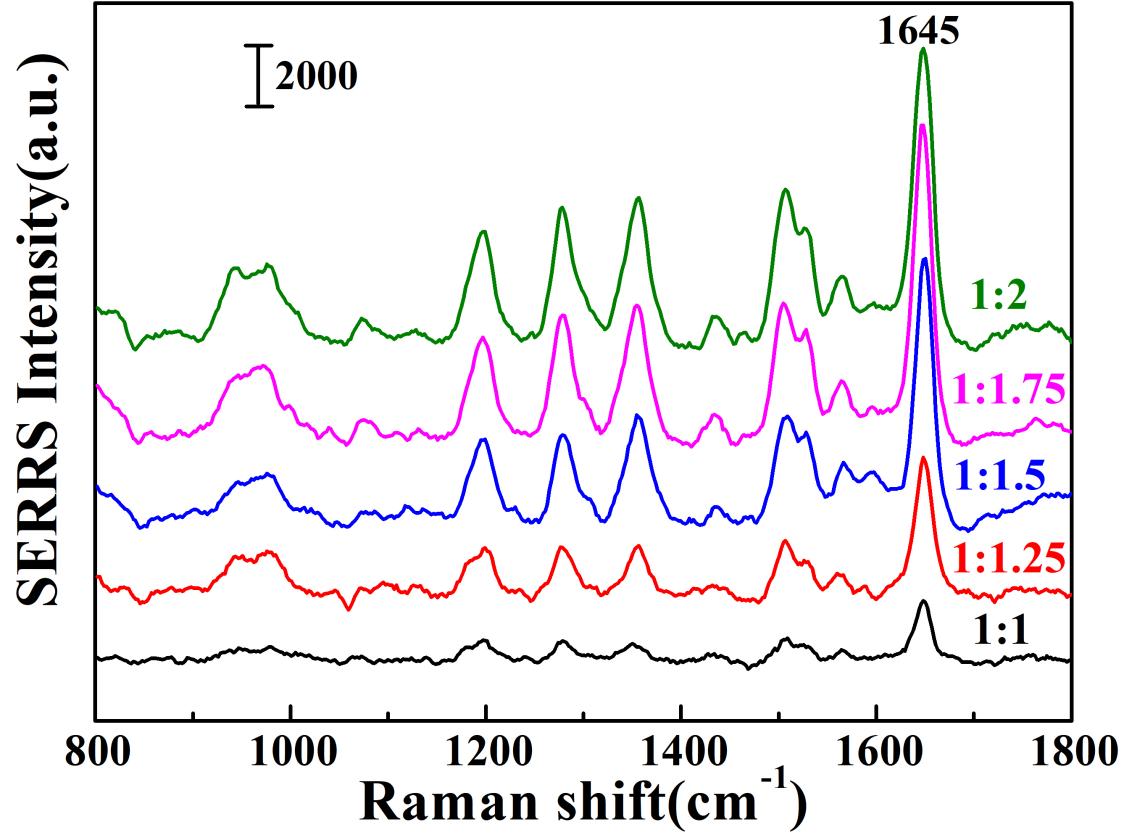
**

**Supplementary Figure 3.** SERS intensity of RBITC at 1645 cm^-1^ of AuNRs different volume ratios (1:1, 1:1.25, 1:1.5, 1:1.75, 1:2) of signal probes and capture probes


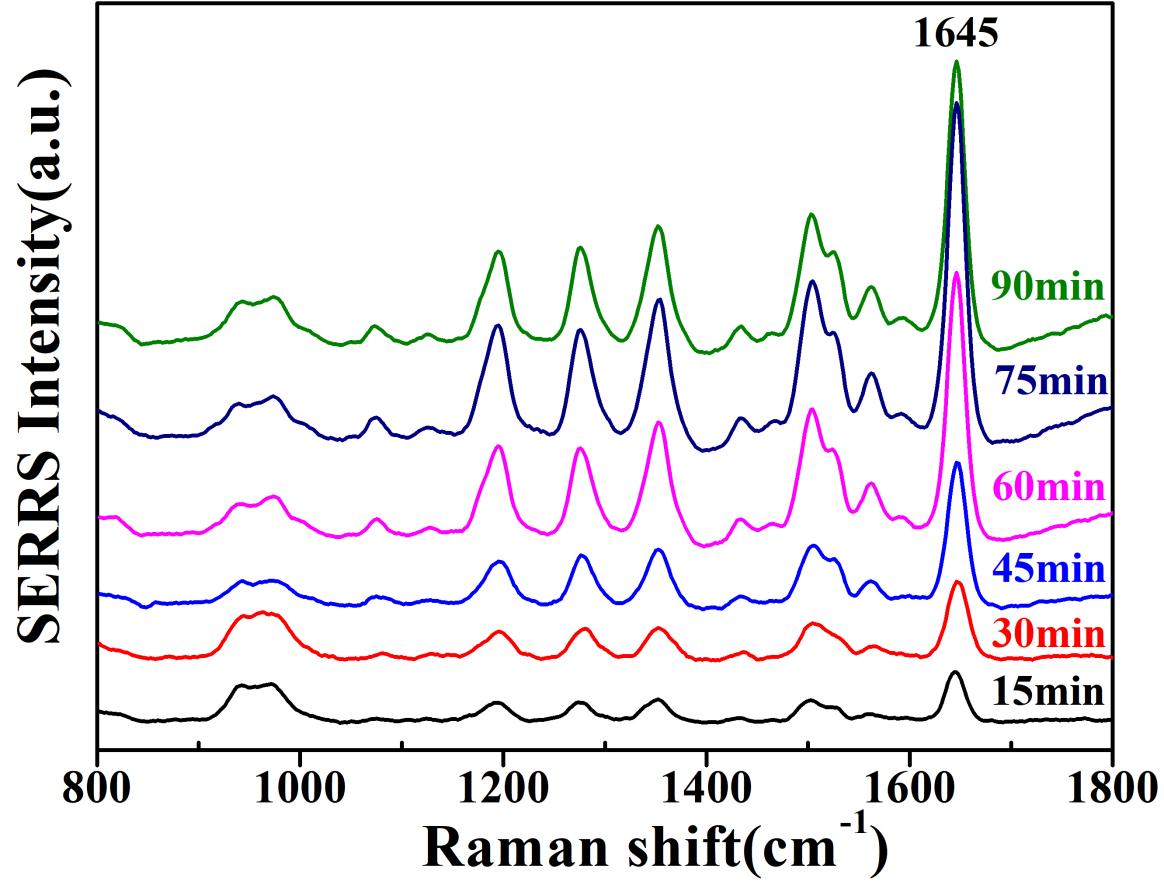


## **Supplementary Figure 4.** SERS intensity of RBITC at 1645 cm^-1^ different incubation times (15, 30, 45, 60, 75, 90 min) of ABA (1×10^-8^ M) added into sensor solution

##
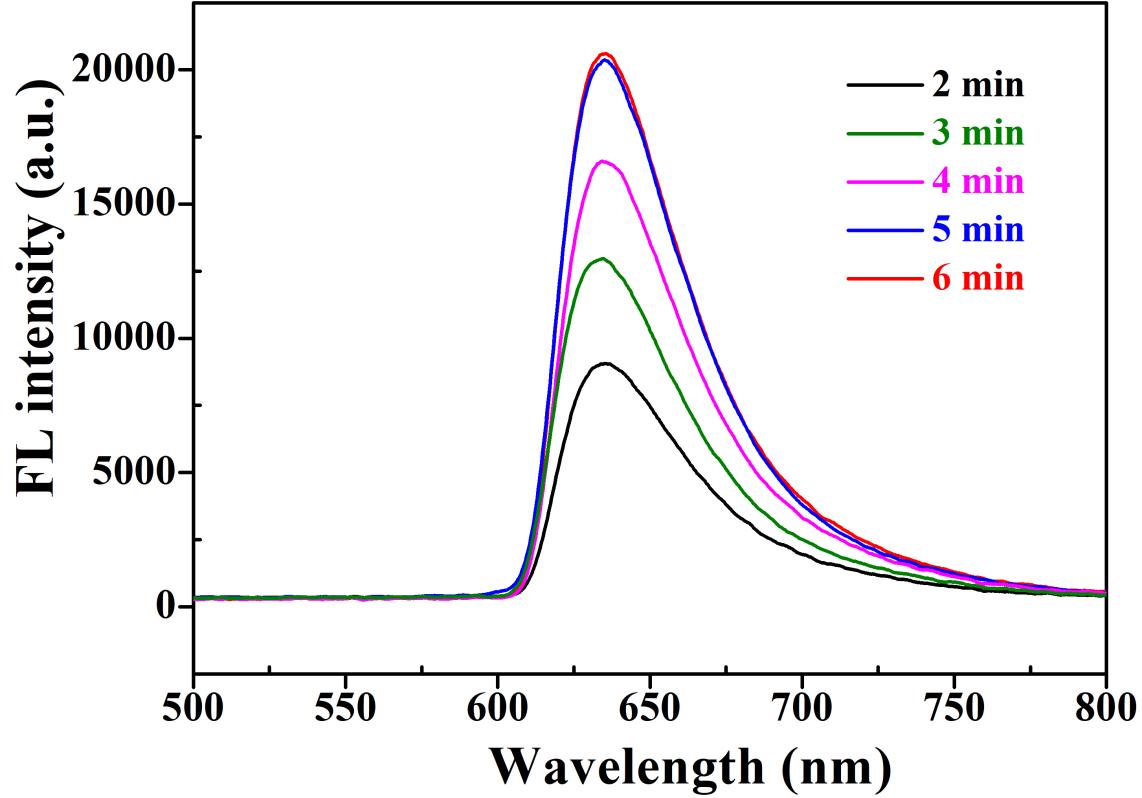


## **Supplementary Figure 5.** FL intensity of RBITC at 639 nm of the sensor solution after etching

**Mechanism of action of etching agent**

The etching agent were composed of 10mM potassium ferricyanide (K_3_Fe[CN]_6_) and 100mM potassium iodide (KI) in a ratio of 1 to 1. The main function of the etching agent was that the weak oxidant K_3_Fe[CN]_6_ oxidizes the Au(0) on the surface of the AuNRs into Au(I), then the Au(I) rapidly combine with I^－^ to generate [AuI]_2-_, which was stable soluble complex and fell off from the surface of the AuNRs and entered the solution. The formation of [AuI]_2-_ results in a greatly reduced concentration of Au(I). After several oxidation-coordination reactions, the AuNRs can be completely dissolved. Supplementary Figure 6 shows the changes of LSPR during the etching of the signal probes. After the etching agent was added into the signal probes, the transverse peak of the AuNRs decreased rapidly and became wider. When the etching time was 5 minutes, the transverse peak of the AuNRs almost disappeared, indicating that the AuNRs had almost been etched in 5 minutes. In addition, the new peaks 301nm and 421nm appeared on the LSPR spectrogram after the addition of etching agent were the SPR peaks introduced by etching agent.

##
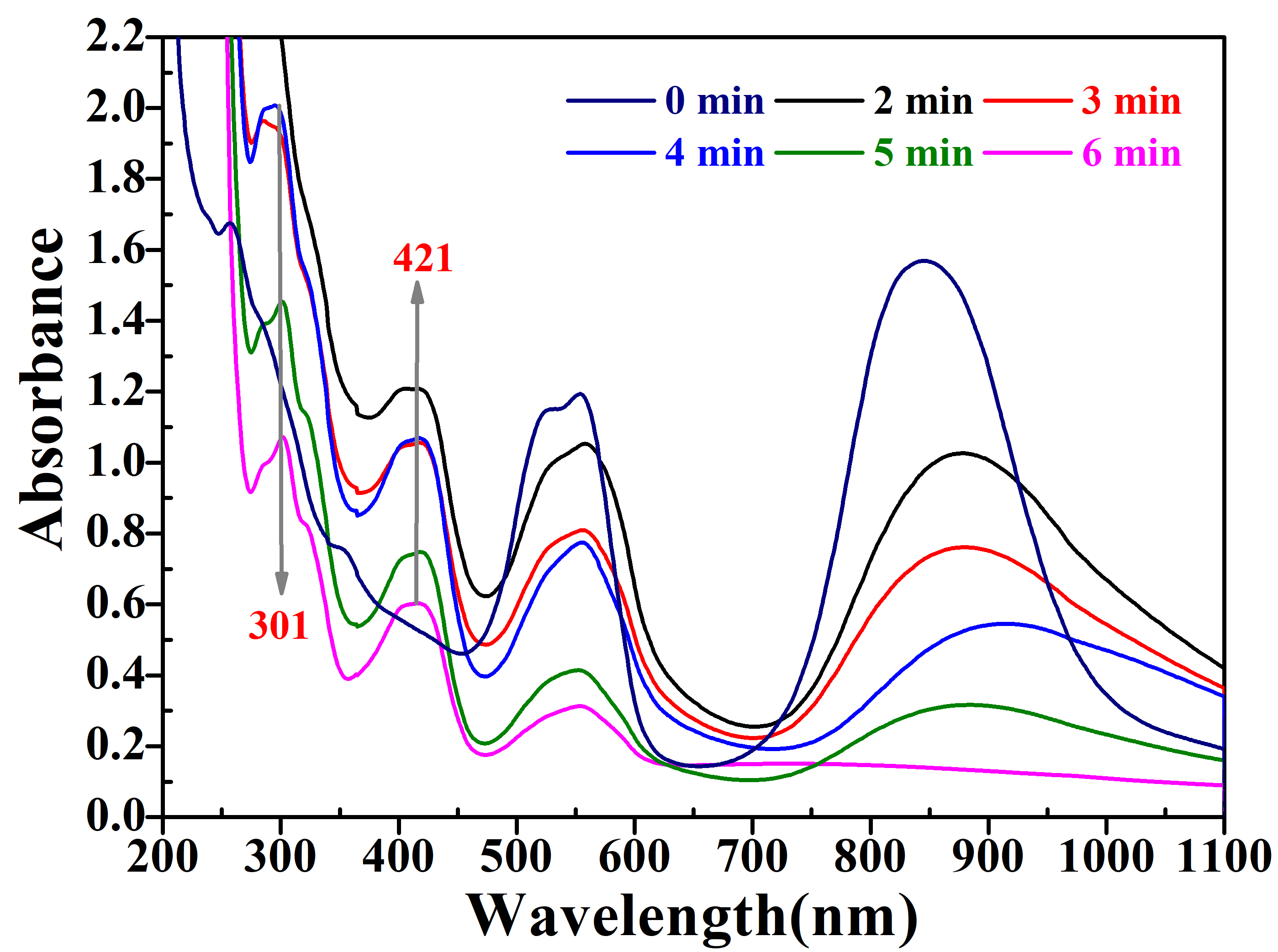


## **Supplementary Figure 6.** The LSPR changes during the etching of the signal probes


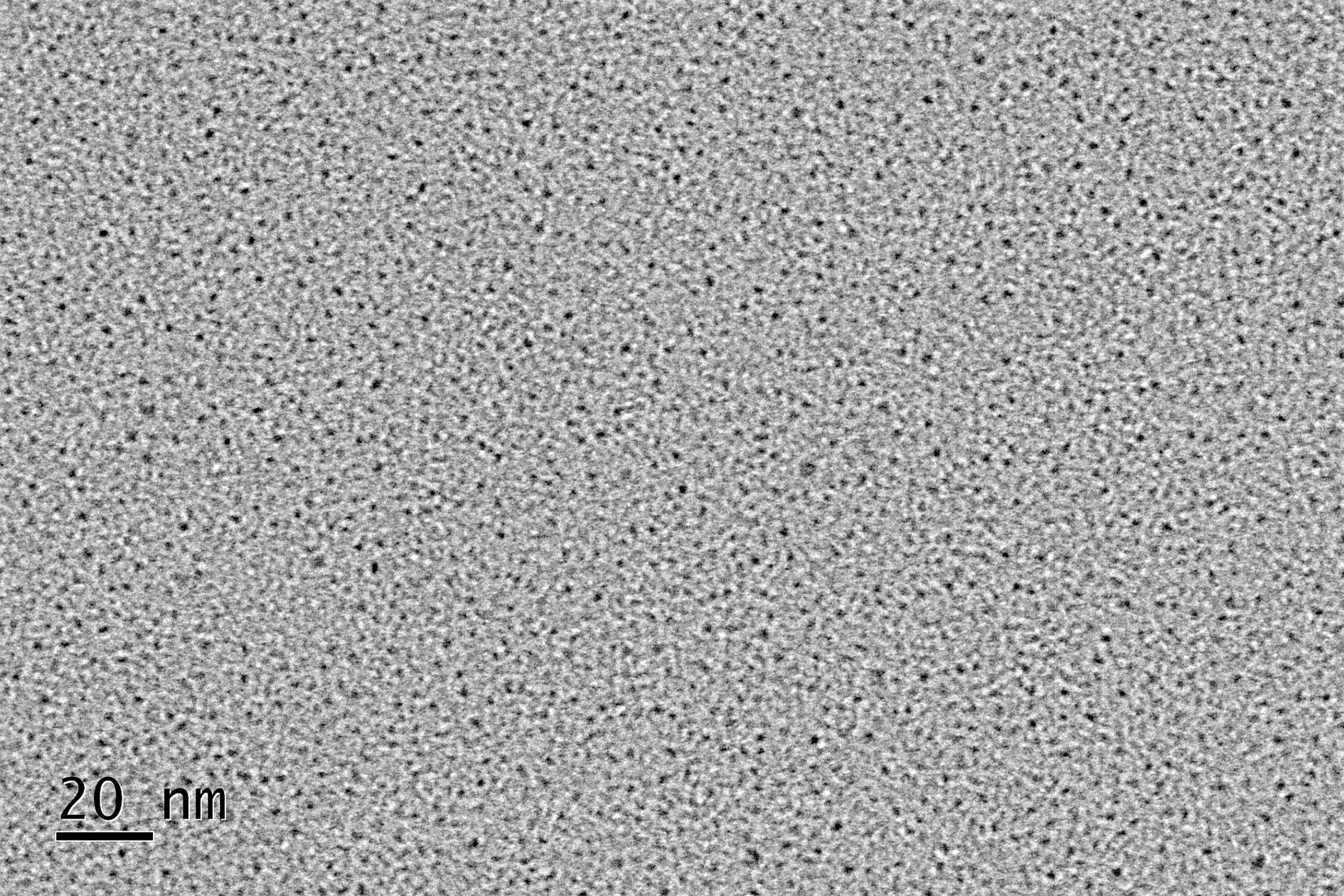


## **Supplementary Figure 7.** The TEM image of etched gold nanorods


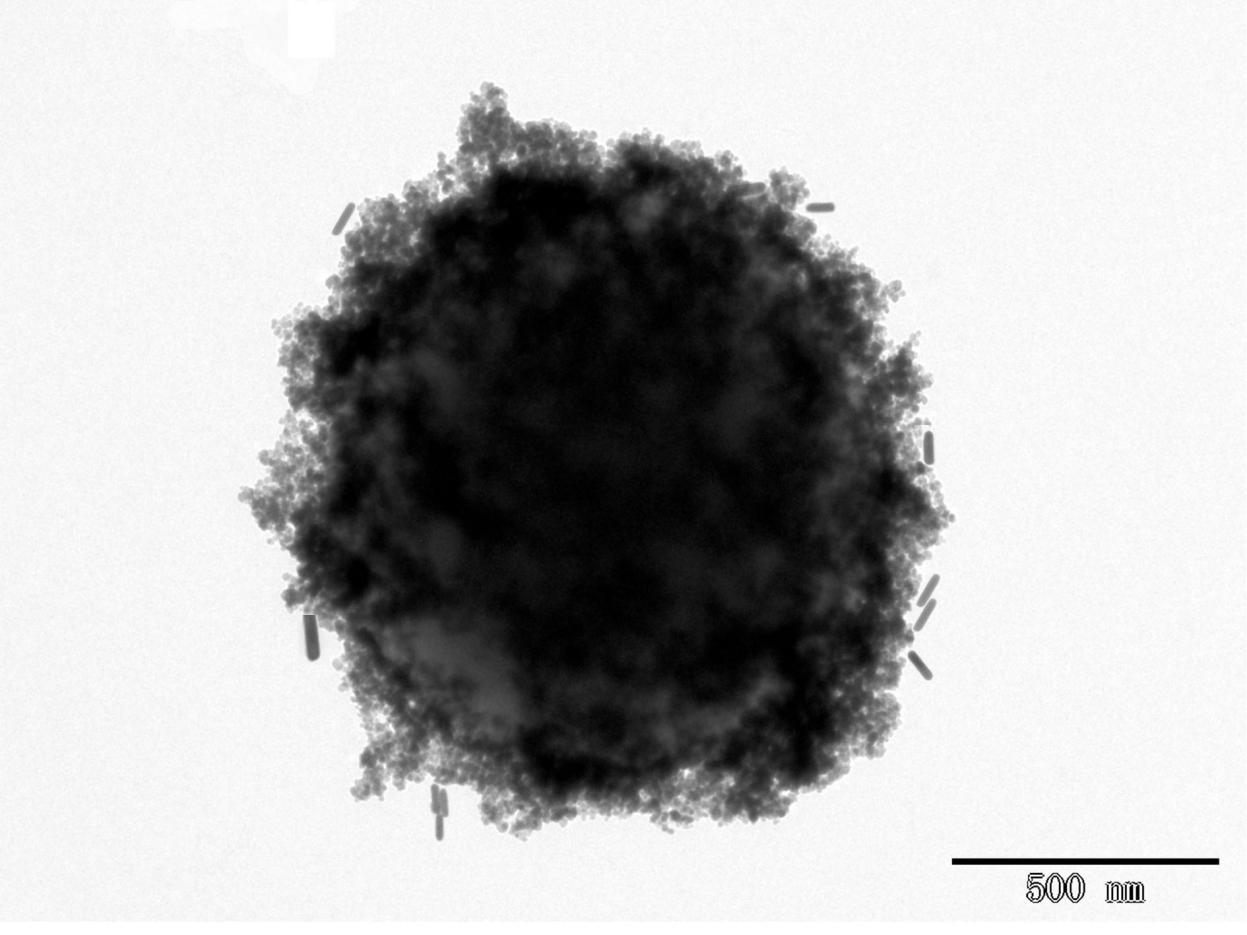


## **Supplementary Figure 8.** The dual-functional aptamer sensor

## **
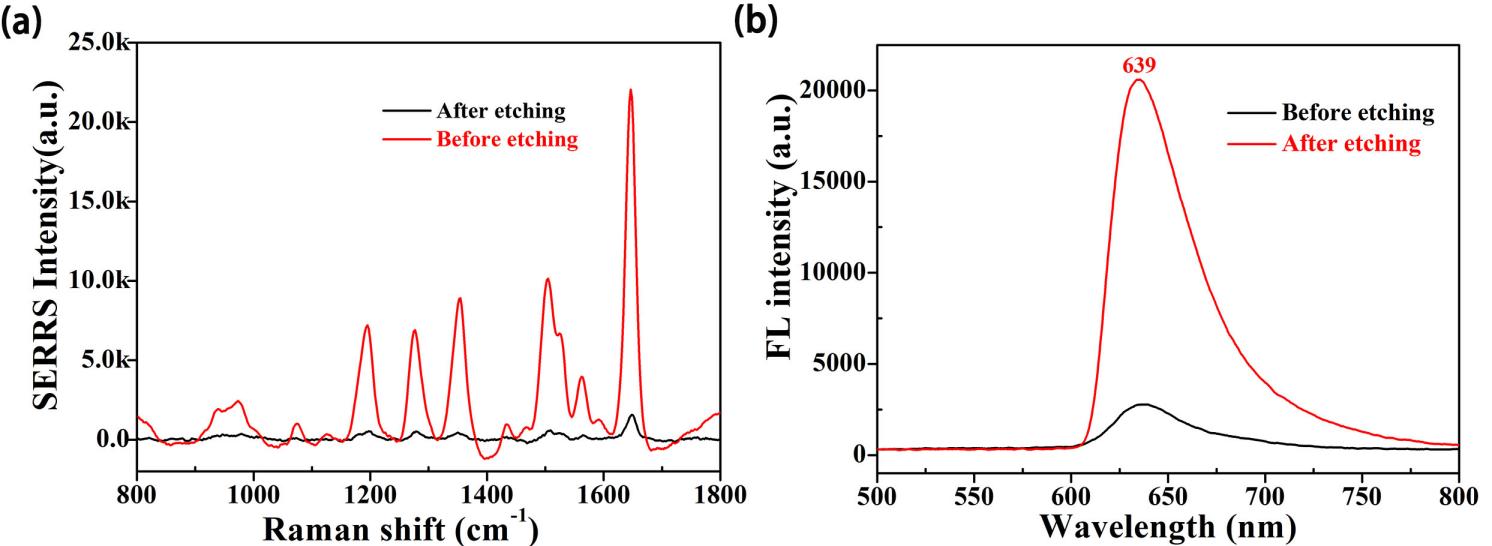
**

## **Supplementary Figure 9.** SERRS and fluorescence intensities of dual-functional aptamer sensor solution before and after etched

##
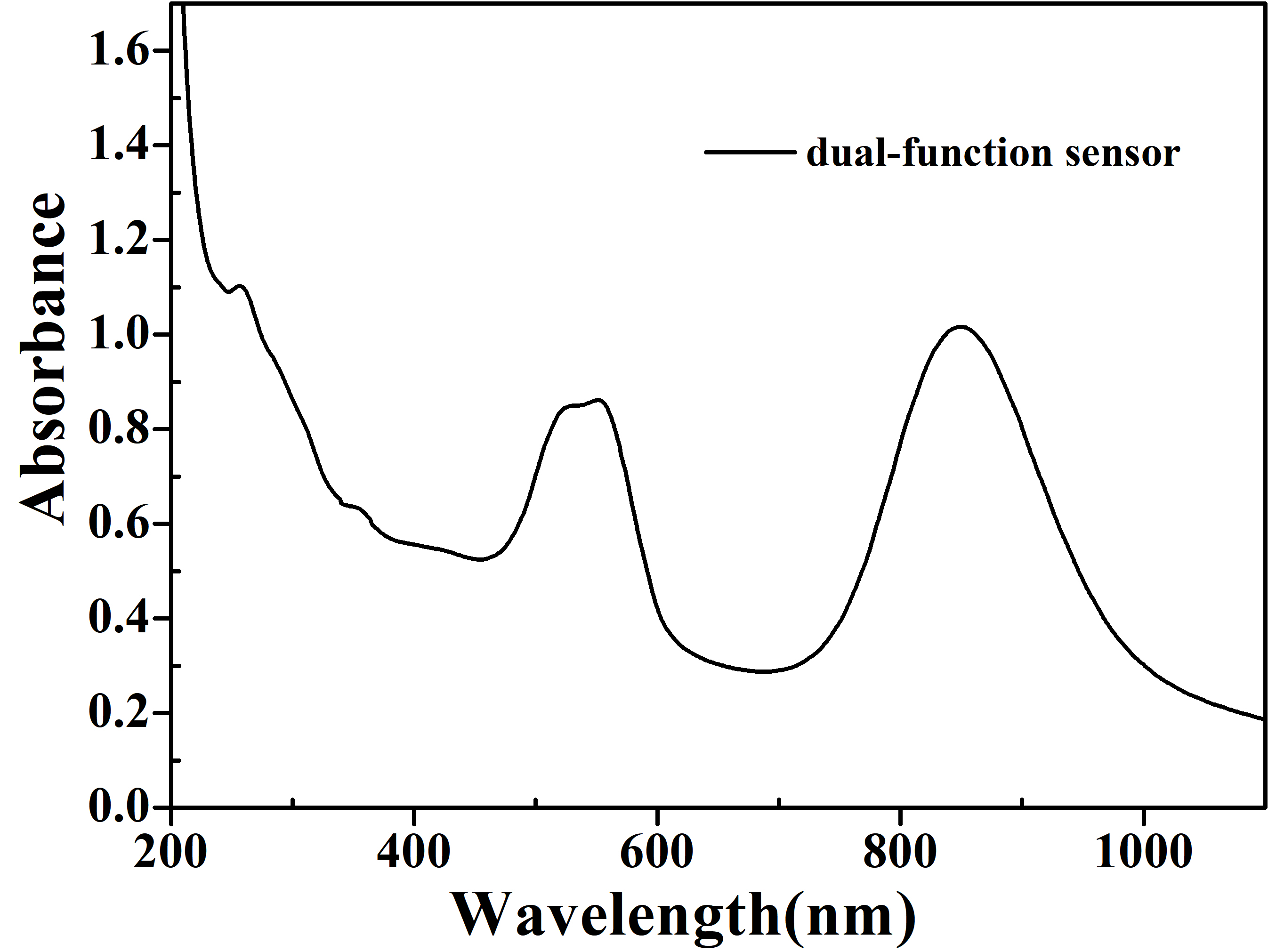


## **Supplementary Figure 10.** The optical UV-Vis spectrum of sensor solution before adding any analyte ABA into solution.
